# Progress Toward Equitable Mpox Vaccination Coverage: A Shortfall Analysis — United States, May 2022–April 2023

**DOI:** 10.15585/mmwr.mm7223a3

**Published:** 2023-06-09

**Authors:** Krishna Kiran Kota, Harrell Chesson, Jaeyoung Hong, Carla Zelaya, Ian H. Spicknall, Aspen P. Riser, Elizabeth Hurley, Dustin W. Currie, R. Ryan Lash, Neal Carnes, Jeniffer Concepción-Acevedo, Sascha Ellington, Ermias D. Belay, Jonathan Mermin

**Affiliations:** ^1^CDC Mpox Emergency Response Team; ^2^Oak Ridge Institute for Science and Education, Oak Ridge, Tennessee.

More than 30,000 monkeypox (mpox) cases were reported in the United States during the 2022 multinational outbreak; cases disproportionately affected gay, bisexual, and other men who have sex with men (MSM). Substantial racial and ethnic disparities in incidence were also reported (*1*). The national mpox vaccination strategy* emphasizes that efforts to administer the JYNNEOS mpox vaccine should be focused among the populations at elevated risk for exposure to mpox (*2*). During May 2022–April 2023, a total of 748,329 first JYNNEOS vaccine doses (of the two recommended) were administered in the United States.^†^ During the initial months of the outbreak, lower vaccination coverage rates among racial and ethnic minority groups were reported (*1*,*3*); however, after implementation of initiatives developed to expand access to mpox vaccination,^§ ^coverage among racial and ethnic minority groups increased (*1*,*4*). A shortfall analysis was conducted to examine whether the increase in mpox vaccination coverage was equitable across all racial and ethnic groups (*5*). Shortfall was defined as the percentage of the vaccine-eligible population that did not receive the vaccine (i.e., 100% minus the percentage of the eligible population that did receive a first dose). Monthly mpox vaccination shortfalls were calculated and were stratified by race and ethnicity; monthly percent reductions in shortfall were also calculated compared with the preceding month’s shortfall (*6*). The mpox vaccination shortfall decreased among all racial and ethnic groups during May 2022–April 2023; however, based on analysis of vaccine administration data with race and ethnicity reported, 66.0% of vaccine-eligible persons remained unvaccinated at the end of this period. The shortfall was largest among non-Hispanic Black or African American (Black) (77.9%) and non-Hispanic American Indian or Alaska Native (AI/AN) (74.5%) persons, followed by non-Hispanic White (White) (66.6%) and Hispanic or Latino (Hispanic) (63.0%) persons, and was lowest among non-Hispanic Asian (Asian) (38.5%) and non-Hispanic Native Hawaiian and other Pacific Islander (NH/OPI) (43.7%) persons. The largest percentage decreases in the shortfall were achieved during August (17.7%) and September (8.5%). However, during these months, smaller percentage decreases were achieved among Black persons (12.2% and 4.9%, respectively), highlighting the need for a focus on equity for the entirety of a public health response. Achieving equitable progress in JYNNEOS vaccination coverage will require substantial decreases in shortfalls among Black and AI/AN persons.

Shortfall analysis, an approach that focuses on the percentage of persons who have not achieved a certain health outcome (*5*), was used to quantify progress in mpox vaccination overall and by racial and ethnic groups. Unlike many conventional disparity measures that compare the rate of a particular health outcome in racial and ethnic minority groups with the rate of another group, such as the group with most favorable rate or the overall population (*7*), shortfall analysis does not require a comparison group. Thus, the shortfall analysis can quantify progress in mpox vaccination coverage for any given racial or ethnic group without regard to changes in vaccination coverage in a comparison group. Further, comparisons of shortfalls across racial and ethnic groups can help to determine if progress in mpox vaccination coverage is equitable. Shortfall in mpox vaccination was calculated as 100% minus the percentage of the eligible population that received a first dose of mpox vaccine; thus, the shortfall measure reflects the deficit in the percentage of vaccinated persons in the eligible population from 100% coverage. Shortfalls, and decreases in shortfalls (measured as a percentage), were calculated at monthly intervals during May 2022–April 2023 for each racial and ethnic group (*6*). A reduction in shortfall indicates progress specific to an individual racial and ethnic group without the need for a reference group, because the reference point is 100% coverage (*6*). Shortfall analyses have been used for measuring progress for a range of health outcomes, such as increases in life expectancies (*6*).

Data on the number of persons aged ≥13 years among seven racial and ethnic groups^¶^ who received a first dose of mpox vaccine were obtained from case surveillance reports submitted to CDC by 49 states, the District of Columbia, and Puerto Rico, during May 2022–April 2023.** The size of the eligible population was calculated as 125% of the sum of the estimated number of MSM with HIV and the estimated number of MSM with HIV preexposure prophylaxis (PrEP) indications. The 25% increase was to account for additional persons who are more likely to be exposed: MSM who are at increased risk for mpox but do not have indications for HIV PrEP; cisgender female, transgender, and gender nonbinary persons who are partners of MSM; close contacts of persons with known or suspected mpox; and persons at risk for occupational exposure to orthopoxviruses (*8*). Estimates of the number of MSM with HIV by race and ethnicity and the estimates of MSM with HIV PrEP indications were obtained using data from CDC Atlas Plus.^††^ To estimate the number of MSM with HIV PrEP indications by race and ethnicity,^§§^ the racial and ethnic distribution of MSM with HIV PrEP indications was assumed to be the same as the racial and ethnic distribution of the male population in the United States.^¶¶^ (*9*). Analyses were conducted using R statistical software (version 4.2.1; R Foundation). This activity was reviewed by CDC and was conducted consistent with applicable federal law and CDC policy.***

Based on review of vaccination data with race and ethnicity reported, an estimated 34.0% of the eligible population had received a first dose of JYNNEOS vaccine by the end of April 2023, corresponding to a calculated shortfall of 66.0% (Table 1). The first dose coverage shortfall was larger among Black (77.9%) and AI/AN (74.5%) persons than among White (66.6%), Hispanic (63.0%), Asian (38.5%), and NH/OPI (43.7%) persons (Figure).

**TABLE 1:** Numbers of persons eligible for JYNNEOS vaccine, cumulative numbers and percentages of persons who received a first dose, and vaccination shortfalls, by race and ethnicity — United States, May 2022–April 2023*

Characteristic/Month	Race and ethnicity^†^							
	Asian	AI/AN	Black or African American	NH/OPI	White	Hispanic or Latino	Multiple races/Other	Total
**Vaccine-eligible population (total)^§^**	**82,103**	**11,171**	**382,876**	**3,116**	**1,036,538**	**419,689**	**60,908**	**1,996,401**
**May–June**								
No. vaccinated	195	8	161	6	1,987	484	132	**2,973**
% vaccinated^¶^	0.2	0.1	0.0	0.2	0.2	0.1	0.2	**0.1**
Shortfall,** %	99.8	99.9	100.0	99.8	99.8	99.9	99.8	**99.9**
**July**								
No. vaccinated	8,879	322	10,762	275	63,016	24,777	4,990	**113,021**
% vaccinated^¶^	10.8	2.9	2.8	8.8	6.1	5.9	8.2	**5.7**
Shortfall,** %	89.2	97.1	97.2	91.2	93.9	94.1	91.8	**94.3**
**August**								
No. vaccinated	33,740	1,466	56,310	1,151	229,852	100,689	23,252	**446,460**
% vaccinated^¶^	41.1	13.1	14.7	36.9	22.2	24.0	38.2	**22.4**
Shortfall,** %	58.9	86.9	85.3	63.1	77.8	76.0	61.8	**77.6**
**September**								
No. vaccinated	43,390	2,196	72,328	1,494	296,611	130,308	31,454	**577,781**
% vaccinated^¶^	52.8	19.7	18.9	47.9	28.6	31.0	51.6	**28.9**
Shortfall,** %	47.2	80.3	81.1	52.1	71.4	69.0	48.4	**71.1**
**October**								
No. vaccinated	47,004	2,511	78,076	1,640	322,104	141,693	34,445	**627,473**
% vaccinated^¶^	57.3	22.5	20.4	52.6	31.1	33.8	56.6	**31.4**
Shortfall,** %	42.7	77.5	79.6	47.4	68.9	66.2	43.4	**68.6**
**November**								
No. vaccinated	48,481	2,654	80,759	1,686	332,859	146,972	35,957	**649,368**
% vaccinated^¶^	59.0	23.8	21.1	54.1	32.1	35.0	59.0	**32.5**
Shortfall,** %	41.0	76.2	78.9	45.9	67.9	65.0	41.0	**67.5**
**December**								
No. vaccinated	49,226	2,724	82,136	1,714	338,297	149,965	36,689	**660,751**
% vaccinated^¶^	60.0	24.4	21.5	55.0	32.6	35.7	60.2	**33.1**
Shortfall,** %	40.0	75.6	78.5	45.0	67.4	64.3	39.8	**66.9**
**January**								
No. vaccinated	49,712	2,760	83,086	1,731	341,663	151,872	37,084	**667,908**
% vaccinated^¶^	60.5	24.7	21.7	55.6	33.0	36.2	60.9	**33.5**
Shortfall,** %	39.5	75.3	78.3	44.4	67.0	63.8	39.1	**66.5**
**February**								
No. vaccinated	50,039	2,785	83,855	1,738	343,751	153,298	37,376	**672,842**
% vaccinated^¶^	60.9	24.9	21.9	55.8	33.2	36.5	61.4	**33.7**
Shortfall,** %	39.1	75.1	78.1	44.2	66.8	63.5	38.6	**66.3**
**March**								
No. vaccinated	50,323	2,822	84,410	1,746	345,438	154,446	37,613	**676,798**
% vaccinated^¶^	61.3	25.3	22.0	56.0	33.3	36.8	61.8	**33.9**
Shortfall,** %	38.7	74.7	78.0	44.0	66.7	63.2	38.2	**66.1**
**April**								
No. vaccinated	50,517	2,850	84,757	1,755	346,599	155,212	37,769	**679,459**
% vaccinated^¶^	61.5	25.5	22.1	56.3	33.4	37.0	62.0	**34.0**
Shortfall,** %	38.5	74.5	77.9	43.7	66.6	63.0	38.0	**66.0**

**Abbreviations**: AI/AN = American Indian or Alaska Native; mpox = monkeypox; MSM = men who have sex with men; NH/OPI = Native Hawaiian or other Pacific Islander; PrEP = preexposure prophylaxis.

* Data from Vermont were not included in the analysis because vaccination data stratified by race and ethnicity were not reported.

^†^ Persons who indicated Hispanic or Latino (Hispanic) ethnicity, regardless of race, were categorized as Hispanic. AI/AN, Asian, Black or African American, NH/OPI, White, or Multiple race (more than one race category selected), or other persons were categorized as non-Hispanic. Persons with missing data on ethnicity or race were categorized as missing or unknown and not included in this analysis.

^§^ The size of the eligible population was calculated as 125% of the sum of the estimated number of MSM with HIV and the estimated number of MSM with HIV PrEP indications. The increase of 25% was to account for all other persons who are more likely to be exposed: MSM who are at increased risk for mpox but do not have indications for HIV PrEP; cisgender female, transgender, and gender nonbinary persons who are partners of MSM; close contacts of persons with known or suspected mpox; and persons at risk for occupational exposure to orthopoxviruses.

^¶^ Cumulative percent vaccinated and shortfalls are rounded to the nearest 10th of a percent.

** Shortfall was calculated by subtracting the cumulative percentage of eligible population that received ≥1 dose of vaccine from 100%.

**FIGURE:**
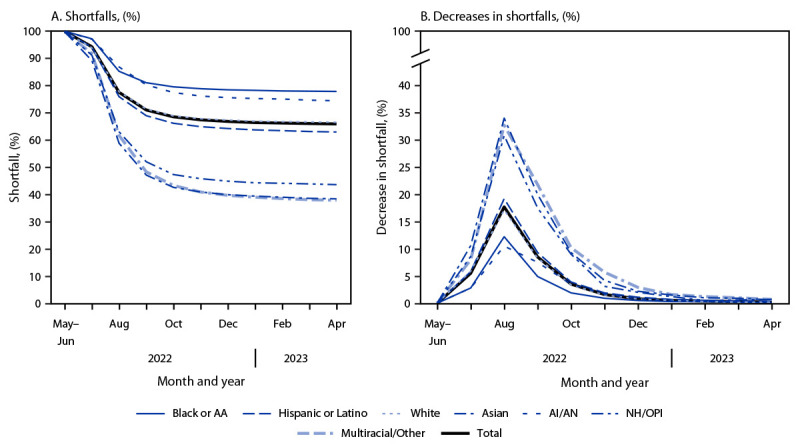
Shortfalls* and percent decreases in shortfalls^†^ in first dose JYNNEOS vaccination, by race and ethnicity^§^ — United States, May 2022–April 2023^¶^ **Abbreviations**: AA = African American; AI/AN = American Indian or Alaska Native; NH/OPI = Native Hawaiian or other Pacific Islander. * Calculated as the difference between 100% vaccination coverage and the reported vaccination coverage. ^†^ Monthly reductions in shortfall were calculated as percent decrease in shortfall compared with the preceding month. Because no vaccines were administered before May, the percent decreases in shortfall for May–June were set to zero. ^§^ Persons who indicated Hispanic or Latino (Hispanic) ethnicity, regardless of race, were categorized as Hispanic. AI/AN, Asian, Black or African American, NH/OPI, White, and Multiple races (more than one race category selected) or other persons were categorized as non-Hispanic. Persons with missing data on ethnicity or race were categorized as missing or unknown and were not included in this analysis. ^¶^ Data from Vermont were not included in the analysis because vaccination data stratified by race and ethnicity were not reported.

From June to July, the overall reduction in shortfall was modest (5.5%), with the smallest reductions observed among Black and AI/AN persons (2.8% each) (Table 2). The largest overall mpox vaccination shortfall reductions were achieved from July to August (17.7%) and August to September (8.5%). However, during these periods, larger shortfall reductions were observed in White (17.1% and 8.3%, respectively), Hispanic (19.2% and 9.3%, respectively), Asian (34.0% and 20.0%, respectively), and NH/OPI (30.8% and 17.5%, respectively) persons, and smaller reductions occurred among Black (12.2% and 4.9%, respectively) and AI/AN (10.5% and 7.5%, respectively) persons. Overall shortfall reductions were smaller from October to November (1.6%), November to December (0.8%), December to January (0.5%), and March to April (0.2%).

**TABLE 2:** Percent reductions in shortfall in administration of first JYNNEOS vaccine doses among vaccine-eligible persons, by race and ethnicity — United States, July 2022–April 2023*

Month	Reduction in shortfall,^†^ %,^§^ by racial and ethnic group^¶^							
	Total	Asian	AI/AN	Black or African American	NH/OPI	White	Hispanic or Latino	Multiple races/Other
July	**5.5**	10.6	2.8	2.8	8.6	5.9	5.8	8.0
August	**17.7**	34.0	10.5	12.2	30.8	17.1	19.2	32.7
September	**8.5**	20.0	7.5	4.9	17.5	8.3	9.3	21.8
October	**3.5**	9.3	3.5	1.9	9.0	3.4	3.9	10.2
November	**1.6**	4.2	1.7	0.9	3.1	1.5	1.9	5.7
December	**0.8**	2.2	0.8	0.5	2.0	0.8	1.1	2.9
January	**0.5**	1.5	0.4	0.3	1.2	0.5	0.7	1.6
February	**0.4**	1.0	0.3	0.3	0.5	0.3	0.5	1.2
March	**0.3**	0.9	0.4	0.2	0.6	0.2	0.4	1.0
April	**0.2**	0.6	0.3	0.1	0.7	0.2	0.3	0.7

**Abbreviations:** AI/AN = American Indian or Alaska Native; NH/OPI = Native Hawaiian or other Pacific Islander.

* Data from Vermont were not included in the analysis because vaccination data stratified by race and ethnicity were not reported.

^†^ Monthly reductions in shortfall were calculated as percent decrease in shortfall compared with the preceding month.

^§^ Reductions in shortfall are rounded to the nearest 10th of a percent.

^¶^ Persons who indicated Hispanic or Latino (Hispanic) ethnicity, regardless of race, were categorized as Hispanic. AI/AN, Asian, Black or African American, NH/OPI, White, and Multiple races (more than one race category selected) or other persons were categorized as non-Hispanic. Persons with missing data on ethnicity or race were categorized as missing or unknown and were not included in this analysis.

## Discussion

During May 2022–April 2023, the shortfalls in receipt of a first dose of mpox vaccine by the eligible population were reduced among all racial and ethnic groups; however, the shortfall was larger among Black and AI/AN persons. The finding of a larger shortfall among Black persons is consistent with the findings of a previous report, which found that the higher vaccination rate among Black males relative to White males (rate ratio = 1.2) was not commensurate with the higher mpox incidence in Black males (rate ratio = 5.8) (*1*). Compared with White persons, Black persons are approximately 20% more likely to be vaccinated (*1*), but they are also approximately 83% more likely than White persons to be members of the vaccine-eligible population. Thus, despite the slightly higher vaccination rate among Black persons,^†††^ the vaccine shortfall is larger among Black persons. The decrease in shortfall was modest from June to July and was most notable in August and September, which could be related to an increase in vaccine supply resulting from the recommendation for dose-sparing intradermal administration of JYNNEOS vaccine on August 9, 2022,^§§§^ and expanded vaccination initiatives, including a focus on addressing disparities in vaccination coverage (*4*). Further, the shortfall among Hispanic persons was less than the overall shortfall, which could also be a consequence of the focus on health equity in the expanded vaccination initiatives (*1*). However, the shortfall reductions were not consistent across all racial and ethnic groups, with smaller reductions among Black and AI/AN populations.

The monthly shortfall reduction is likely a more meaningful measure of progress toward equity in vaccination coverage than is the increase in vaccination coverage, because the former quantifies progress toward 100% mpox vaccination, and the latter can be biased against racial and ethnic groups with lower coverage (i.e., the same percentage point increase in coverage will result in a larger relative percent increase among groups with lower coverage than in groups with higher coverage) (*5*). Accordingly, the decline in shortfall is consistent with the principle of proportional justice, in which equitable progress toward 100% vaccination requires that racial and ethnic minority groups with larger vaccination shortfalls achieve larger percentage point increases in vaccination coverage than do groups with smaller shortfalls (*5*,*6*). Focusing on the racial and ethnic groups with larger mpox vaccination shortfalls and prioritizing resources and improving access to vaccination for these groups can reduce the overall shortfall in mpox vaccination while simultaneously promoting health equity.

The findings in this report are subject to at least three limitations. First, data on race and ethnicity were missing for 9% of vaccine recipients; if vaccinated persons with missing race and ethnicity data were included in the analysis, the overall shortfall would be 62.5% rather than 66.0% (*8*). Second, the estimated sizes of racial and ethnic groups constituting the vaccine-eligible population are uncertain (*8*). For example, the racial and ethnic distribution of MSM with HIV PrEP indications is unknown; for this analysis, this distribution was assumed to be the same as the that of the U.S. male population, based on the evidence of similar levels of HIV PrEP indications across racial and ethnic groups (*9*). However, the actual racial and ethnic disparities in mpox vaccination shortfall could be notably higher than estimated if there are racial and ethnic disparities in the distribution of MSM with HIV PrEP indications^¶¶¶^ (*10*). Finally, estimates of the size of the vaccine-eligible population were increased by 25% to include additional groups (other than MSM with HIV or MSM with HIV PrEP indications) who might benefit from vaccination. However, the need to expand the size of the vaccine-eligible population might be more pronounced for racial and ethnic minority groups because of social determinants of health and sexual partnership selection patterns based on race and ethnicity (e.g., persons tending to choose sexual partners of same race and ethnicity, age, and other characteristics). Accordingly, the vaccination shortfalls might be underestimated, particularly for some racial and ethnic minority groups.

This mpox vaccination shortfall analysis provides a better understanding of progress in mpox vaccination in eligible populations by racial and ethnic groups than assessing increases in percentage of persons vaccinated. The shortfall reductions among Black and AI/AN persons were smaller than the overall shortfall reductions at all monthly intervals considered, leading to larger and persistently higher shortfalls in these groups compared with overall shortfall. A focus on achieving equal reductions in shortfalls is needed to achieve equitable progress in mpox vaccination coverage. Effective strategies could include engaging trusted messengers, community-based organizations, and providers in the design and delivery of vaccination messages using relatable cultural and language nuances to reach populations at increased risk for mpox.**** To minimize the risk for future mpox outbreaks, vaccination coverage among all eligible persons needs to increase substantially, particularly among racial and ethnic minority groups with the largest shortfalls.

**Summary**

**What is already known about this topic?**

Vaccination efforts during the 2022 U.S. monkeypox (mpox) outbreak focused on populations at elevated risk for acquiring mpox.

**What is added by this report?**

As of April 2023, two thirds (approximately 66.0%) of mpox vaccine–eligible persons remained unvaccinated. The shortfall (difference between 100% coverage and reported first-dose coverage) was largest among Black or African American (Black) persons (77.9%). The largest monthly decreases in overall shortfall were in August (17.7%) and September (8.5%). However, during these months, smaller shortfall reductions were achieved among Black persons (12.2% and 4.9%, respectively).

**What are the implications for public health practice?**

Vaccination coverage among racial and ethnic minority groups with the largest shortfalls needs to increase substantially to reduce disparities in vaccination coverage and increase health equity.

## Footnotes

* https://www.cdc.gov/poxvirus/mpox/interim-considerations/overview.html (Accessed May 11, 2023).

†
https://www.cdc.gov/poxvirus/mpox/response/2022/vaccines_data.html (Accessed May 14, 2023).

§
https://www.cdc.gov/poxvirus/mpox/health-departments/vaccine-equity-pilot.html

¶ Persons who indicated Hispanic ethnicity, regardless of race, were categorized as Hispanic. AI/AN, Asian, Black, NH/OPI, White, or Multiple race (more than one race category selected) or other persons were categorized as non-Hispanic. Persons with missing data on ethnicity or race were categorized as missing or unknown and not included in this analysis.

** Data from Vermont were not included in the analysis because vaccination data stratified by race and ethnicity were not reported.

†† The estimated number of MSM with HIV by race and ethnicity was obtained from jurisdiction-specific estimates of 2020 HIV prevalence by race and ethnicity from Atlas Plus (an interactive tool that allows for the creation of customized tables, maps and charts using CDC’s HIV, hepatitis, sexually transmitted infections, and tuberculosis surveillance data) describing men with HIV whose HIV transmission category was male-to-male sexual contact or male-to-male sexual contact and injection drug use. https://www.cdc.gov/nchhstp/atlas/index.htm

§§ The number of MSM with indications for HIV PrEP was estimated as the ratio of the jurisdiction-specific number of MSM receiving HIV PrEP and the jurisdiction-specific HIV PrEP coverage from CDC’s Atlas Plus. The number of MSM with indications for HIV PrEP by race and ethnicity was estimated by multiplying the number of MSM with HIV PrEP indications by the race and ethnicity weights derived from the racial and ethnic distribution of the male population in the United States. https://wonder.cdc.gov/single-race-v2021.html

¶¶ This approach was employed based on the evidence indicating that the proportion of MSM reporting HIV PrEP indications was similar across racial and ethnic groups. A supplemental analysis was conducted in which the racial and ethnic distribution of MSM with HIV PrEP indications was assumed to be the same as the racial and ethnic distribution of new HIV diagnoses in MSM using data from CDC’s Atlas Plus.

*** 45 C.F.R. part 46, 21 C.F.R. part 56; 42 U.S.C. Sect. 241(d); 5 U.S.C. Sect. 552a; 44 U.S.C. Sect. 3501 et seq.

††† The probability that a Black person would be in the vaccine-eligible population was calculated as the number of Black persons in the vaccine-eligible population divided by the number of Black persons aged ≥13 years in the United States (382,876 / 34,715,303 = 0.011), using 2021 population estimates from CDC wonder (https://wonder.cdc.gov/single-race-single-year-v2021.html). Using the same approach, the probability that a White person would be in the vaccine-eligible population was calculated as 1,036,538 / 171,591,284 = 0.006. The relative likelihood of a Black person being in the vaccine-eligible population compared with a White person was 1.83 (0.011 / 0.006).

§§§ https://www.fda.gov/media/160774/download#:~:text=The%20FDA%20has%20granted%20an,high%20risk%20for%20monkeypox%20infection

¶¶¶ In the supplemental analysis in which the racial and ethnic distribution of MSM with HIV PrEP indications was assumed to be the same as the racial and ethnic distribution of new HIV diagnoses in MSM, the shortfalls in vaccination as of April 2023 for the three largest racial and ethnic groups were Black, 87.8% (versus 77.9% in the main analysis); Hispanic, 72.0% (versus 63.0%); and White, 44.4% (versus 66.6%). However, the overall shortfall was the same in both analyses (66.0%).

****
https://www.cdc.gov/poxvirus/mpox/resources/toolkits/equity.html
